# Delayed hydrocephalus after excision of a colloid cyst: a case report

**DOI:** 10.1186/s13256-022-03453-0

**Published:** 2022-06-07

**Authors:** Logan Gray, Nathan Quig, Myungsa Kang

**Affiliations:** 1 Department of Anesthesiology, University of North Carolina, N2198, CB #7010, Chapel Hill, NC 27599-7010 USA; 2 Department of Neurosurgery, University of North Carolina, Chapel Hill, NC USA

**Keywords:** Colloid cyst, Delayed hydrocephalus, Case report

## Abstract

**Background:**

In this case report we describe an unusual case of a patient who underwent resection of a colloid cyst and then presented 6 weeks postoperatively with obstructive hydrocephalus. There appear to be no prior reports of such a delayed complication after colloid cyst resection.

**Case presentation:**

A 50-year-old Caucasian woman underwent resection of a colloid cyst with an uncomplicated perioperative course. Postoperative imaging demonstrated complete resection of the cyst. She was discharged home on postoperative day 4 but presented 6 weeks later with symptoms of obstructive hydrocephalus resulting in poor neurologic outcome and ultimately death.

**Conclusion:**

Patients presenting with symptoms of hydrocephalus after resection of a colloid cyst should be followed closely, and timely placement of an external ventricular drain may be critical.

## Introduction

Colloid cysts are benign tumors that typically occur in the foramen of Monro. They are relatively rare with an incidence cited as 3.2 million cases per year, which is about 2% of all intracranial tumors [1]. They are thought to be neuroepithelial in origin, made up of a single layer of mucin-producing epithelium with a fibrous outer layer and filled with a gelatinous material. Although they are histopathologically benign, these tumors may cause mass effect, which rarely can result in severe hydrocephalus and even death. Over half of all patients with colloid cysts have symptoms associated with hydrocephalus, including headache, nausea/vomiting, unsteady gait, and mental status changes [1]. The hydrocephalus is typically related to the tumor blocking drainage of cerebrospinal fluid (CSF) through the foramen of Monro [2]. The risk of rapid clinical deterioration is estimated to be 3–35%, and the associated risk of death in this group has been calculated to be 5–38% [1].

Written Health Insurance Portability and Accountability Act authorization and informed consent was obtained from the patient’s spouse. This article adheres to the applicable Enhancing the Quality and Transparency of Health Research (EQUATOR) guidelines.

## Case description

**Fig. 1:**
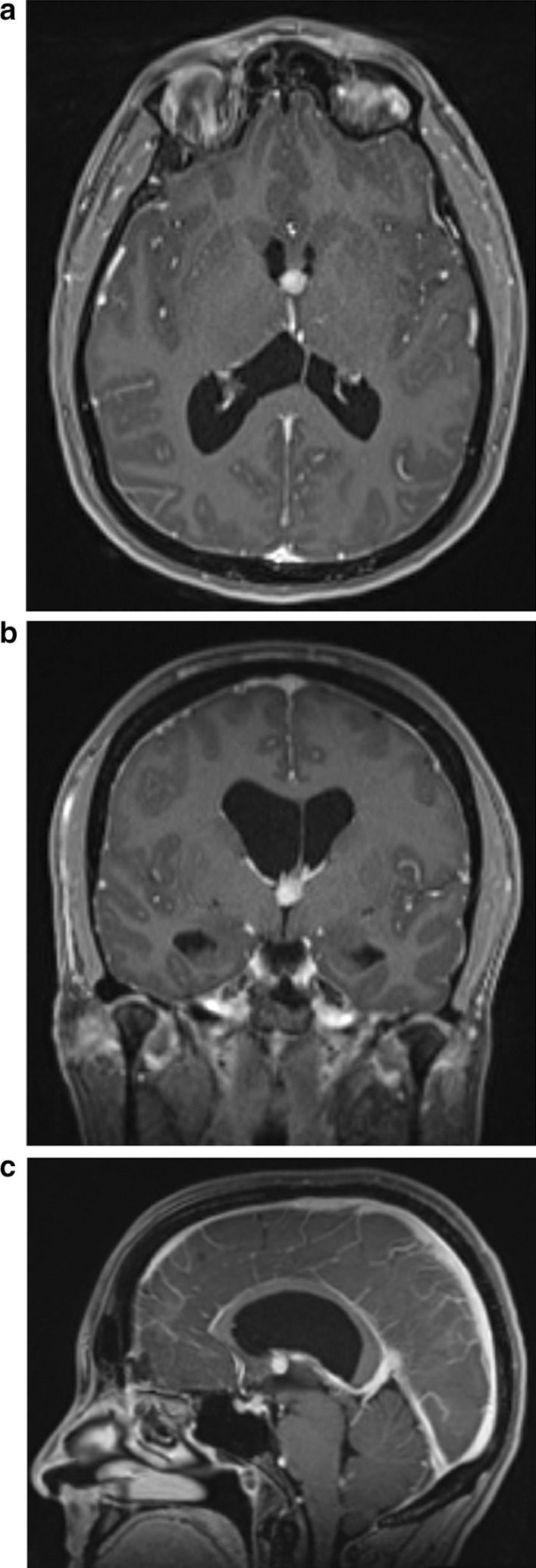
Preoperative axial (**a**), coronal (**b**), and sagittal (**c**) T1 postcontrast magnetic resonance imaging demonstrating a colloid cyst at the foramen on Monroe. Note the dilated lateral ventricles and bowing of the corpus callosum suggestive of chronic hydrocephalus

We present the case of a patient who underwent craniotomy for resection of a colloid cyst and then presented 6 weeks later with obstructive hydrocephalus. A 50-year-old Caucasian woman with past medical history otherwise significant only for hypothyroidism presented with symptoms of intermittent headaches and unsteadiness. Imaging revealed a colloid cyst (7 × 9 × 8 mm^3^), and the patient elected to undergo craniotomy for resection (Fig. 1).

**Fig. 2:**
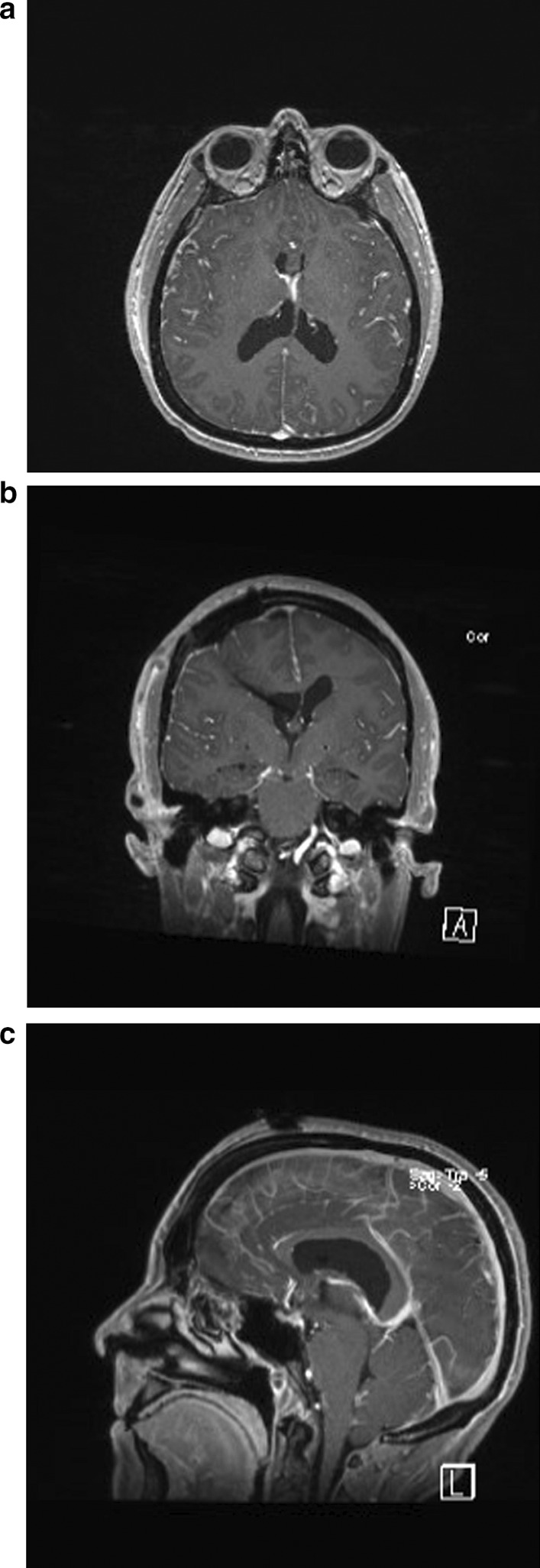
Postoperative axial (**a**), coronal (**b**), and sagittal (**c**) T1 postcontrast magnetic resonance imaging demonstrating complete resection of the cyst with mild ventriculomegaly and small amount of intraventricular hemorrhage

The patient underwent transcortical microscopic resection of her colloid cyst with intraoperative computed tomography (CT) image guidance and placement of external ventricular drain. Intraoperatively, the intracranial pressure (ICP) was noted to be mildly elevated upon entering the dura. The cyst, which appeared to be most densely adherent to the choroid plexus of the roof of the third ventricle, was successfully resected. The patient’s intraoperative course was unremarkable, and she was extubated in the operating room (OR) prior to transfer to the neurosurgical intensive care unit (NSICU). Postoperative imaging was read as consistent with complete resection of the cyst with some mild ventriculomegaly and small-volume intraventricular hemorrhage (Fig. 2).

Her postoperative course was largely unremarkable as well, although she did report some short-term memory impairment prior to discharge. At her follow-up visit 2 weeks after the surgery, she reported that she was doing well with rehabilitation, although she continued to report memory issues. She had another follow-up visit a few weeks later, at which she again expressed concern about memory issues, as well as difficulty with spelling, anxiety, and insomnia.

**Fig. 3:**
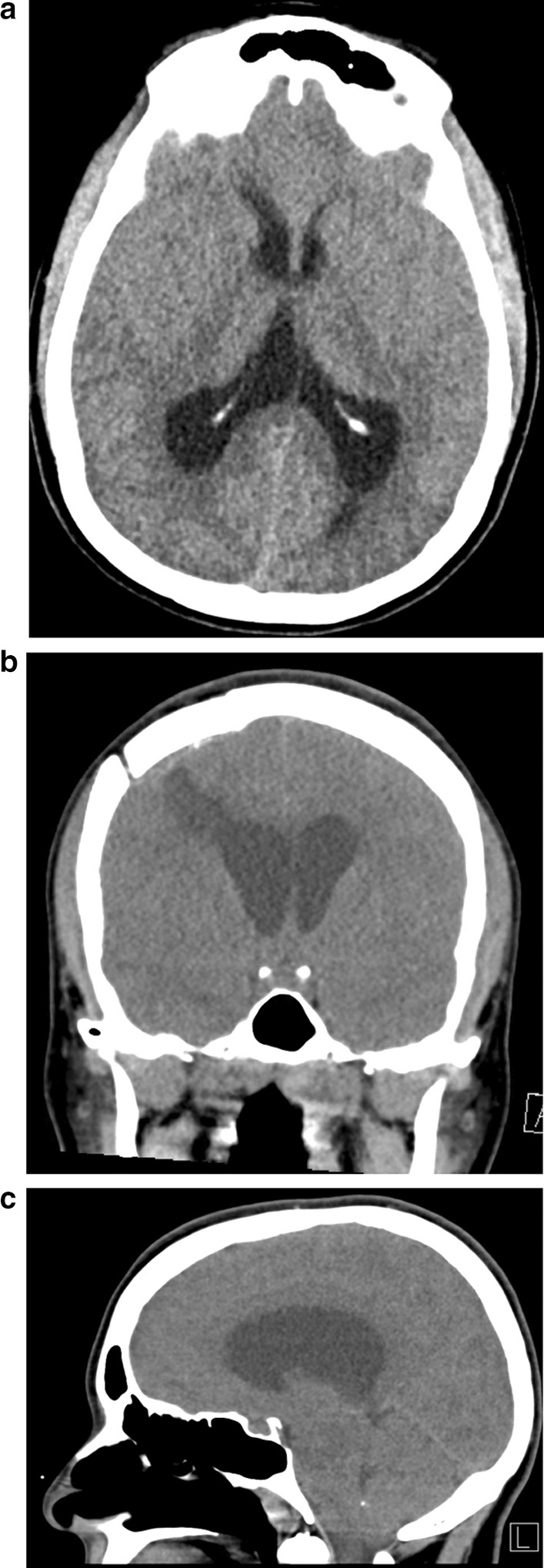
Axial (**a**), coronal (**b**), and sagittal (**c**) CT scan demonstrating resection of the colloid cyst and dilatation of the lateral ventricles on presentation to the ED 6 weeks postoperatively. Note that the third and fourth ventricles are not dilated, suggestive of obstructive hydrocephalus

She presented to the emergency department (ED) 6.5 weeks postoperatively after falling with complaints of headache and nausea/vomiting. On initial examination, the patient was drowsy but oriented and able to follow commands. Head CT showed worsening hydrocephalus (Fig. 3), and the patient was admitted for external ventricular drain (EVD) placement and intracranial pressure (ICP) monitoring. While awaiting transfer to the NSICU, the patient became acutely obtunded with decerebrate posturing and dilated pupils. The patient was intubated and stabilized, and an EVD was placed in the ED prior to transfer to the NSICU. She returned to the OR for a ventriculoperitoneal shunt (VPS) several days later, which was uneventful from an anesthetic and surgical perspective. Unfortunately, the patient’s neurologic prognosis remained poor, with imaging of the brain consistent with ischemic hypoxic encephalopathy. She was transferred to a skilled nursing facility and subsequently died.

## Discussion

There are numerous case reports in literature of sudden unexpected coma and death in patients with colloid cysts. A 21-year-old patient developed symptoms of hydrocephalus while jogging and within hours became comatose [3]. Head CT demonstrated a hemorrhagic cyst with obstructive hydrocephalus. Air travel has been noted to be related to poor outcomes in patients with colloid cysts as well, possibly caused by the varying altitudinal pressure in the cabin [4, 5]. Another patient developed symptoms of severe hydrocephalus after riding on a roller coaster [6]. The authors theorized that the rapid acceleration and deceleration might result in transient hydrocephalus that could lead to collapse and concluded that these patients should be advised to avoid such high-velocity activities. While these cysts are typically not symptomatic in children, cases of sudden death have been reported in children as well. A 13-year-old who presented with hydrocephalus and underwent a diagnostic lumbar puncture (LP) rapidly deteriorated soon afterwards. The authors conclude that the LP in the setting of symptoms of hydrocephalus may have precipitated herniation and recommend avoiding LP in patients with hydrocephalus [7].

A case series of 65 patients suggests that symptoms associated with sudden death in patients with colloid cysts include sudden severe headaches and vomiting [8]. Other features that were noted to be associated with sudden death include larger cysts >1 cm and associated hemorrhage, as well as radiographic evidence of ventriculomegaly [8]. The neurosurgeons placed an external ventricular drain in this patient because of the relatively large size of her cyst. In one retrospective study, the risk of needing a VPS shunt postoperatively was nearly 13 times higher in patients with cysts larger than 6 mm [9].

There have also been several reports of perioperative complications in patients undergoing colloid cyst excision. One patient developed fatal acute disseminated encephalomyelitis after resection of a colloid cyst with ultrasonic aspirator [10]. A patient who underwent endoscopic resection of a colloid cyst developed a posterior fossa hematoma that resulted in hydrocephalus and neurologic compromise [11]. There have also been reports of patients developing hemiparesis and vasospasm postoperatively [12, 13].

Ultimately, it is unclear what caused this patient’s severe hydrocephalus 6 weeks after an apparently successful resection of her colloid cyst. Some possible etiologies include postsurgical edema or granulation and scar tissue at the foramen of Monroe, causing delayed obstructive hydrocephalus. It is also possible that her mild Chiari malformation in combination with chronic hydrocephalus caused her acute decline. Her presentation was felt to be quite unusual as chronic delayed hydrocephalus typically follows a more insidious neurologic decline.

## Conclusion

While there are numerous case reports of sudden coma and death in patients with colloid cysts at initial presentation or in the immediate perioperative period, there appear to be no prior cases reported with the complication of severe hydrocephalus occurring several weeks after the initial resection. This patient’s postoperative imaging indicated complete resection of the cyst, so it is unclear what caused her delayed obstructive hydrocephalus. Patients presenting with symptoms of hydrocephalus after resection of a colloid cyst should be followed closely, and timely placement of an EVD may be critical.

## Abbreviations

CSF: Cerebrospinal fluid
ED: Emergency department
NSICU: Neurosurgical intensive care unit
OR: Operating room
VPS: Ventriculoperitoneal shunt
